# Periodic protein adsorption at the gold/biotin aqueous solution interface: evidence of kinetics with time delay

**DOI:** 10.1038/srep36232

**Published:** 2016-11-03

**Authors:** H. Neff, H. M. Laborde, A. M. N. Lima

**Affiliations:** 1 Universidade Federal de Campina Grande, Center for Electrical Engineering and Informatics, Department of Electrical Engineering, Campina Grande, Paraiba, Brazil; 2 Universidade Federal de Campina Grande, Center for Science and Technology, Department of Chemical Engineering, Campina Grande, Paraiba, Brazil

## Abstract

An oscillatory molecular adsorption pattern of the protein neutravidin from aqueous solution onto gold, in presence of a pre-deposited self assembled mono-molecular biotin film, is reported. Real time surface Plasmon resonance sensing was utilized for evaluation of the adsorption kinetics. Two different fractions were identified: in the initial phase, protein molecules attach irreversibly onto the Biotin ligands beneath towards the jamming limit, forming a neutravidin-biotin fraction. Afterwards, the growth rate exhibits distinct, albeit damped adsorption-desorption oscillations over an extended time span, assigned to a quasi reversibly bound fraction. These findings agree with, and firstly confirm a previously published model, proposing macro-molecular adsorption with time delay. The non-linear dynamic model is applicable to and also resembles non-damped oscillatory binding features of the hetero-catalytic oxidation of carbon monoxide molecules on platinum in the gas phase. An associated surface residence time can be linked to the dynamics and time scale required for self-organization.

Non-damped periodic immobilization features have been reported for numerous adsorption systems. The phenomenon is linked to conditions, where more than a single reactant is involved in the adsorption process. A well known example in the gas phase is the hetero-catalytic oxidation of CO to CO_2_ on (110) oriented platinum surfaces at elevated temperature, in presence of oxygen. Specific conditions, at which these phenomena occur, were first identified in the Nobel prize winning work of G. Ertl^1^. Electro-catalytically driven oscillations also have been recorded at the Pt-aqueous electrolyte interface for various organic molecules that are usable as oxidation reactants, especially in fuel cell applications^2^,^3^. These initially poorly understood effects were occasionally noticed before, but wrongly attributed to experimental flaws. Such systems can be described by a set of coupled rate equations for each reactant with concentration *u*_1,2,…,*n*_:


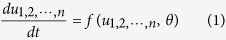


where *θ* is the surface coverage, and *f*(*u*_1,2,…,*n*_, *θ*) describes the reaction through associated external control and kinetic parameters. Under certain conditions, and varying with the complexity of the reaction, non-linear differential equations emanate, which can be solved numerically. Depending on selected parameters, this may lead to oscillations, or instability and even chaotic behavior, connected to spatial modulations and processes of self-organization.

For macro-molecular, especially protein binding onto solid surfaces, in absence of specific surface reactions, such effects have yet not been observed. Nonetheless, macro-molecular film formation at solid-liquid interfaces remains complex and is of relevance for understanding inter-cell signaling, metabolism, drug activity, biocompatibility in connection with medical implantation and transplantation methods, immune response mechanisms and immunoassay based biosensors. Adsorption is driven by electrostatic/electrochemical, van-der-Waals, Lewis acid/base, hydrogen bonding and covalent/chemical interaction forces. It varies with all: pH of the solvent/buffer, substrate material, hydrophobic or hydrophilic surface conditions, ionic strength, protein type and molecular structure, concentration, temperature and externally biased or inherent electrochemical potentials across the metal-liquid interface^4^,^5^,^6^. Due to their iso-electronic point (pI) of 6.5 in PBS buffer, neutravidin (NAv) molecules are weakly negatively charged. A weak repulsive interfacial force is acting onto the proteins.

Despite of research work over more than half century, the detailed binding mechanisms are still not fully understood and application of available adsorption models remained controversial^7^. As a characteristic feature, the maximum achievable surface coverage in the high concentration limit approaches a single monolayer (ML) in case of Langmuir type adsorption processes^8^,^9^ and its generalized models^10^. Alternatively, the so-called random-sequential-adsorption (RSA) mechanism^11^,^12^ and improved versions^13^,^14^,^15^,^16^ thereof have been invoked which, in contrast, propose a significantly reduced maximum surface coverage under equilibrium conditions. It is defined by the so-called jamming limit *θ*_*JL*_, placed slightly above 0.5 ML for disc like molecules and varies somewhat with the molecular shape and dimension.

Virtually all experiments of single protein adsorption displayed a steady temporal increase of the surface coverage *θ*(*t*) and/or film thickness, along with a declining growth rate *dθ*(*t*)/*dt*. Immobilization from solution typically exhibits two different adsorption states, assigned as weakly reversible and (chemically) stronger attached irreversible fractions^17^. Recently, a adsorption model that differs considerably from the Langmuir approach (LA), known as adsorption kinetics with time delay, has been proposed^18^,^19^. Unlike the classical LA that features steady exponential temporal variations of the kinetic quantities, it proposes coverage overshoot and an oscillatory pattern. The model has been invoked to explain the temporal evolution of the surface tension of gelatin solution at the water-air interface. The associated temporal evolution, shown in Fig. 5 of ref. 19, and recorded over an exceptionally long period of time exhibits rather weak modulations. Until today, the model still lacks direct experimental proof for protein adsorption at the solid-liquid interface. A compilation of six currently exploited adsorption models, along with experimental data, is outlined in Fig. 1. Generally, LA-type binding (type I), random sequential adsorption (RSA)-type mechanisms (type II) and associated refined models typically exhibit a smooth temporal decline, comprise a maximum (type IV) or remain constant (type III) over limited period of time. None of these adsorption mechanisms exhibits oscillations in the adsorption transients. Experimental evidence, as illustrated by adsorption transients *θ*(*t*) and growth rates *dθ*(*t*)/*dt* for bovine serum albumin (BSA), avidin and human serum (h.Serum), which is a protein mixture, onto the plain gold surface are illustrated in Fig. 1c–e^20^. It is worth to note that the surface coverage of avidin-as well as NAv - after removal of the reversibly attached fraction- approaches a single monolayer (ML), as is predicted by the LA. Differently, the final state surface coverage of irreversibly attached h.Serum and BSA protein layers approximately corresponds to 0.5 ML, supporting the RSA mechanism. However, in the initial phase, the experimentally obtained adsorption characteristics of all protein solutions is best described by the type IV mechanism from ref. 10, which considers macromolecular diffusion, among other features. In contrast, the theoretically more demanding time delay (type V) and non-linear catalytic adsorption models (type VI) from Fig. 1b exhibit oscillatory kinetic features.

An attempt has been made in this work to identify a macro-molecular adsorption system, comprising oscillatory kinetic features. Real time surface Plasmon resonance (SPR) recording in the angular interrogation mode at high temporal resolution has been employed for assessment of the molecular surface coverage and associated growth rates. This feature is considered an appropriate kinetic parameter, applicable to all adsorption systems and model independent. The gold-biotin-neutravidin multi-layer arrangement under consideration is an important adsorption system, utilized in the design of a popular class of biosensors that relies on biotinylated substrates and/or adsorbents, known as the avidin-biotin complex^21^.

## Adsorption kinetics with time delay

The concept of adsorption kinetics with time delay originates from an earlier published theory that treated solute transport through a cell membrane. Time delay was introduced to explain the occasionally observed phenomenon of overshoot and oscillatory permeation in biological cell systems. The same idea was applied to the adsorption kinetics of solutes from the solution phase onto a solid surface. For technical details of this theoretical approach we refer the reader to refs 18 and 19, and references cited therein. Macro-molecules with complex structures and dissolved in solution, may accumulate on a solid substrate or the air-solution interface differently from the classical Langmuir and its refined models. For the reverted process, attached particles may require a certain time span for desorption from the interface, as a result of their conformational changes or other surface processes that affect the residence time. This accounts for a time delay *τ* in the temporal evolution of the solute concentration at the interface, possibly leads to overshoot and oscillation, preferably at high solute concentration.

Briefly, the temporal evolution of the solute concentration *C*(*t*) and the number of particles present at the surface *N*(*t*) is described by two kinetic equations, comprising a delay time *τ: J* is the net flux of diffusing molecules from the solution onto the surface. *v*_*a*_ and *v*_*d*_ are the adsorption and desorption velocities, respectively and *k*_*a*_ and *k*_*d*_ the associated rate constants.


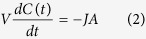



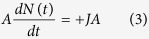


with *J* = *v*_*a*_ − *v*_*d*_, and *v*_*a*_ = *k*_*a*_*C*(*t*)[*N*_*m*_ − *N*(*t*)], and


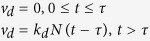


As *C*(*t*) and *N*(*t*) satisfy the conservation relation


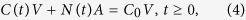


this implies


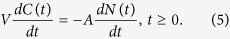


Upon the introduction of three constants *α* = *k*_*a*_*C*_0_, *K* = (*k*_*a*_/*k*_*d*_)*C*_0_ and *R* = *N*_*m*_*A*/(*C*_0_*V*), equations (6) and (7) are extracted. *C*_0_ is the initial bulk solute concentration, *V* is the solution volume, *R* is the ratio of maximum amount of solutes adsorbed onto the surface to the total solute amount and is of the order of one. *K* and *α* is the ratio (*k*_*a*_/*k*_*d*_) and product, respectively, of the (association/dissociation) binding constants with *C*_0_, and


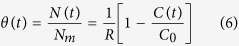


is the surface coverage; *A* is the active surface area; *N*_*m*_ is the number available binding sites. Based on (6) one may derive the following dynamic model for *θ*(*t*):


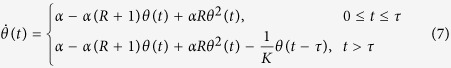


This model is non-linear, and the temporal evolution of surface coverage, *θ*(*t*), conveys information regarding the time delay, and the kinetic constants *k*_*a*_ and *k*_*d*_.

## Experimental

Sulfo-NHS-S-S-biotin (ThermoFisher Scientific), as well as free biotin, BIo (Fluka) and NAv (ThermoFisher Scientific) were dissolved in phosphate buffered saline (PBS-buffer) solution at pH = 7.23, utilizing consecutive attachments with bi-layer formation to the metal film at moderate solute concentration (C_*s*_). Initial biotin binding to the metal substrate leads to a full, self-assembled mono-layer (SAM), comprising a molecular size and film thickness of 1 nm. Experimental details and data extraction methods to obtain the surface coverage are outlined elsewhere^9^. The molecule is also known as vitamin H and is widely used in biochemical research as a molecular label in the assay design of various types of biosensors. Dissolved biotin molecules are not electrically charged and electrostatic gold surface-biotin or BIo-NAv interactions are absent. Well-developed Biotin SAM formation requires presence of an initially hydrophilic Gold surface and Biotin concentrations at the solubility limit in ethanol-water solutions. Biotin coverage values slightly exceeding 1 ML most likely originate from weak physisorptive effects and presence of intermolecular forces.

Briefly, neutravidin is the de-glycosylated form of the protein avidin. The carbohydrate fraction is removed from the molecule; the nearly spherical molecular structure of the protein remains largely unchanged. The molecular size and film thickness is 5.8 nm. This globular protein is best known for its ability to quasi covalently bind up to four biotin molecules^21^, with binding pockets located within the macro-molecular volume. Adsorption transients were differentiated for time *t*, to extract the growth rate. Two different NAv-solutions have been exploited: i) freshly prepared protein solutions, using sealed vials with lypholized powder material; ii) aged prepared protein using the same solution, but after freeze storage for 3.7 years at a temperature range of −5 °C to 0 °C, and exposed to several freeze-thaw cycles over this long period. The adsorption characteristics of biotin onto these protein films in terms of achievable coverage and growth rates, when generated from this aged solution, differ considerably from fresh prepared ones. Aqueous protein solutions are highly vulnerable to organic contaminations through, e.g., spores, algae, and bacteria. They cause rather fast degradation with loss of function through fouling. Commercial suppliers of protein solutions, especially glycoproteins (antibodies), hence usually add a low concentration (0.1%) of sodium azide (NaN_3_). This toxic ionic compound stabilizes the solution over longer period of time, if kept at low temperature, but reliable storage periods are usually not provided. It is worth to mention that this treatment was not applied here.

Optical polymeric SPR-biochips have been employed in the present work. The sensing spot is covered with a magnetron sputtered 50 nm thin, optically semi-transparent gold film. The SP-output signal provides the adsorption induced (effective) refractive index (RIU) variation with time Δ*n*_eff_(*t*), from which the surface coverage can be extracted^9^. The achievable accuracy <*δθ*> corresponds to ≈0.03 ML. Adsorption experiments were performed in a temperature controlled laboratory environment at 23 ± 0.2 °C. The output signal is low noise, long term stable and comprises a low instrumental drift value of −3 × 10^−7^ RIU/min. All, the chip fabrication technology, the optical set-up for SPR operation in the angular interrogation mode at wavelength of 670 nm, along with the micro-fluidic flow cell set-up and physical/technical aspects of the related SPR sensing method are described in detail elsewhere^22^. Figure 2 illustrates the pertaining instrumental temporal stability. The SPR output signal of the refractive index variation Δ*n*_eff_(*t*) at the gold-water interface was recorded over a time period of 1250 s. The upper curve indicates the pertaining instrumental temperature. The lower black line illustrates the instrumental stability in Δ*n*_eff_(*t*), absence of oscillations, with a drift value < −1 × 10^−6^ RIU/min. Figure 2b,c demonstrate the temporal evolution of coverage *θ*(*t*) and adsorption rate *dθ*(*t*)/*dt* for NAv (aged and fresh), as well as associated immobilization features of free biotin (there is no aging effect) onto the respective protein layers beneath, comprising a (reverted) gold metal-NAv-biotin adsorption system. This system corresponds to the adsorption of NAv onto a plain Gold metal film, followed by the adsorption of Biotin onto the NAv pre-layer. The degraded protein material accounts for an approximately factor two smaller adsorption rate *dθ*(*t*)/*dt* for biotin, compared to the fresh protein material, and a factor 5 lower surface coverage of biotin to the NAv film beneath. In contrast, the initial NAv adsorption rate of the degraded NAv solution is a factor 7 higher, when compared with the fresh material. In the former case, LA type adsorption seems to dominate (*θ* approaches 1 ML), the fresh solution rather agrees with the random sequential adsorption mechanism (RSA, the jamming limit approaches 0.55 ML). Oscillations in *θ*(*t*) as well as adsorption rates are not observed.

## Results and Discussion

**Figure 1:**
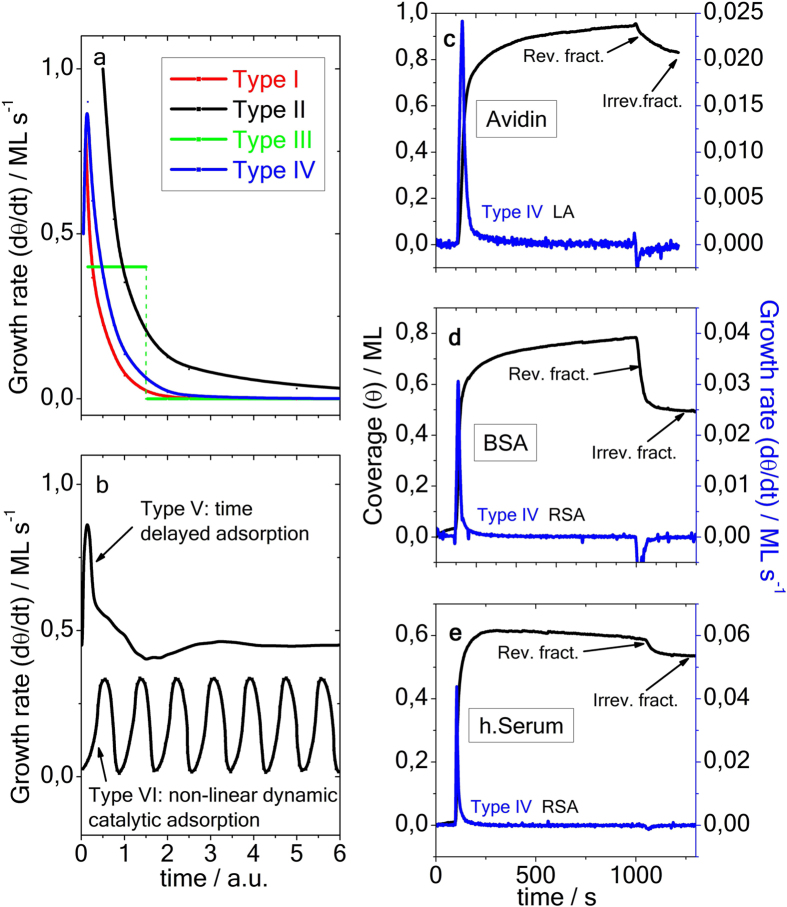
Sketch of the temporal evolution of growth rates *dθ*(*t*)/*dt* for 6 different molecular binding models (**a,b**) and experimentally obtained protein adsorption transients for avidin, BSA and human Serum (**c–e**)^20^, as indicated in the figure. Type I: Irreversible Langmuir type binding^8^; type II: Random sequential (irreversible) adsorption (RSA)^12^; type III: generalized refined RSA type models^13^,^14^,^15^,^16^; type IV: the generalized molecular kinetic model^10^; type V: the kinetic model with time delay^18^,^19^; type VI: catalytic non-linear dynamic model^1^,^2^,^3^.

**Figure 2:**
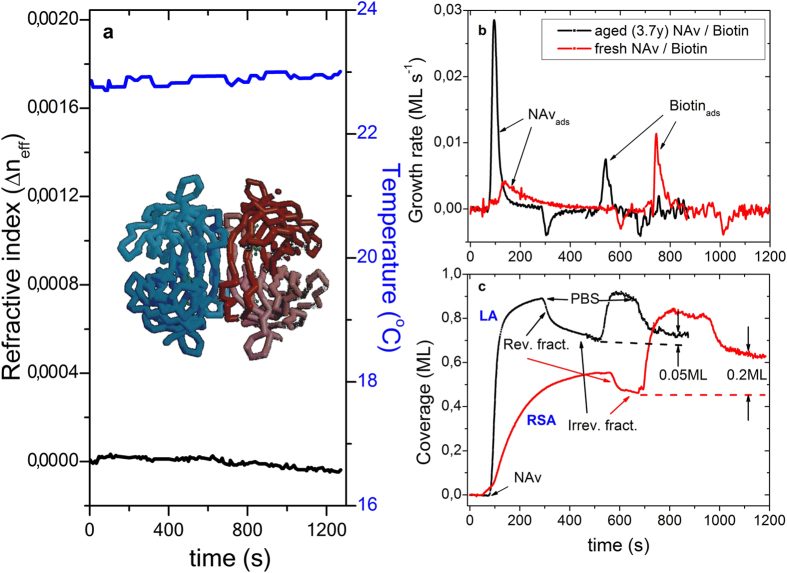
SPR output signal Δ*n*_eff_(*t*) for the temporal evolution and instrumental stability at the Au-water interface (lower black plot), in absence of an adsorption film and recorded over time period of 1250 s. The inset to (**a**) exhibits a molecular structure model of the avidin-biotin complex taken from Fig. 4A of ref. 21. The biotin molecules (green) are located inside at four symmetric pockets. The upper curve displays the instrumental temperature variation. Reverted adsorption system [Au-NAv-Biotin]: NAv and free biotin coverages *θ*(*t*) and sticking coefficients *dθ*(*t*)/*dt* for fresh and aged NAv solutions in (**b,c**) respectively.

**Figure 3:**
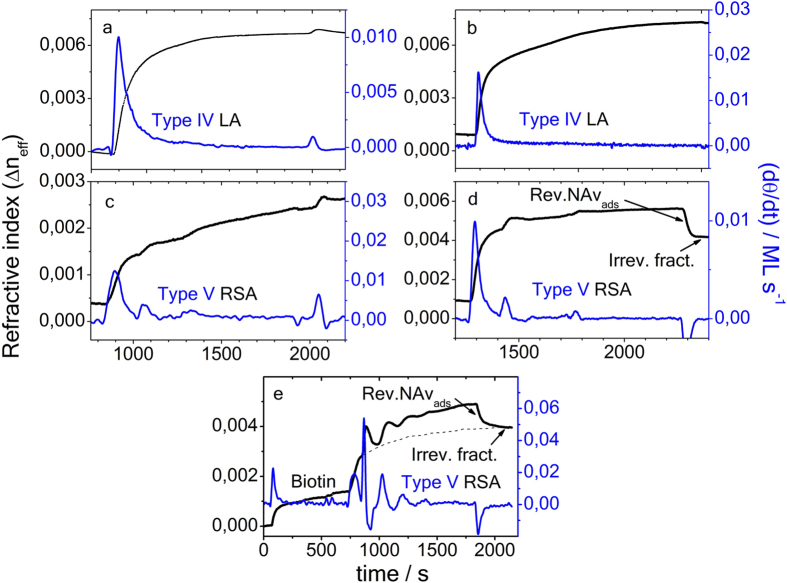
Experimentally recorded SPR adsorption transients of NAv of the effective refractive index variation (left scale) and extracted growth rate *dθ*(*t*)/*dt*. Plot recorded in absence of pre-adsorbed sulfonated biotin onto a plain gold film (**a**); in presence of approximately 1 ML biotin and admission of a degraded NAv-solution (**b**); in presence of approximately 0.2 ML free biotin (**c**); recording repeated after extended period, using fresh prepared NAv solution and BIo-SAM coverage ≈0.9 ML(**d**); and in presence of approximately 1.2 ML sulfonated BIo-SAM in (**e**). NAv bulk concentration *C*_0_ = 0.3 *μ*mol/L. The broken line in (**e**) indicates a steady background signal of slow irreversible NAv attachment.

Figure 3a–e display the temporal evolution of effective adsorption induced refractive index variation Δ*n*_eff_(*t*) and growth rates *dθ*(*t*)/*dt* (lower blue lines) and influence of various experimental conditions, upon admission of NAv solution at moderate C_*s*_ to plain and biotin pre-covered gold surface. Figure 3a is recorded in absence of the BIo-SAM, but the initial time of NAv adsorption begins near 900 s instead of 100 s in Fig. 2c. As said before, Δ*n*_eff_(*t*) scales proportional with the coverage. A smooth steady increase in *θ*(*t*) and Δ*n*_eff_(*t*), respectively, are resolved under these conditions in Fig. 3a. The initial temporal variation of the protein growth rate is ascribed to the adsorption mechanism of type IV from Fig. 1a. The final state is in accordance with the LA, comparable to avidin from Fig. 1c. In Fig. 3b, the gold surface is pre-covered with a biotin film, comprising coverage close to a mono-layer, but aged and chemically slightly degraded NAv solution was admitted to the surface. The resulting protein growth rate and film coverage are similar to those from Fig. 3a. There is no oscillation visible. In Fig. 3c, the metal film is pre-covered with an incomplete, approximately 0.2 ML thin sulfonated biotin film, and already exhibits weak modulations during NAv attachment, as are clearly resolved in the growth rate *dθ*(*t*)/*dt*. Figure 3d illustrates the effect of admission fresh prepared macromolecular adsorbent, with the free BIo-SAM coverage maintained slightly below 1 ML. In contrast to Fig. 3b, oscillations are well resolved. In presence of a sulfonated biotin film, slightly above mono-layer thickness (≈1.2 ML) on the gold surface in Fig. 3e, pronounced, albeit attenuated modulations are resolved over the admission period. The time period between maxima initially is 190 s, and decreases to 180 s. At *t* = 1700 s, plain PBS buffer solution is admitted and removes the loosely attached (quasi-reversible) protein fraction. This leaves an incomplete NAv film, irreversible attached to the biotin film beneath, comprising a self-limited coverage to not more than 0.5 ML. Appropriate adsorption mechanisms are assigned in the growth rate plot: an initial homogeneous, complete BIo-SAM forms in Fig. 3e at *t* ≤ 200 s, followed by irreversible adsorption of NAv onto the biotin layer beneath at *t* = 700 s and subsequent adsorption-desorption oscillations. These superimpose with a slow steady background increase of coverage. Most likely, it is due to slow and continuous irreversible binding of a small NAv fraction.

The oscillations likely originate from NAv binding to excess biotin molecules (≈0.2 ML), followed by delayed desorption, due to extended surface residence time. The individual steps are indicated in the figure. In earlier experiments, we have observed in ref. 9 that biotin films can grow in solution up to three mono-layers. However, the outer two biotin layers are reversibly attached and easily removed by buffer rinse.

The quality of the materials involved, as well as carefully performed initial surface cleaning procedures and protocols, plays a crucial role in the adsorption process and possible biotin-neutravidin complex formation. As is illustrated in Fig. 2b,c, the interaction between NAv and biotin is considerably compromised for the degraded/aged protein solution.

**Figure 4:**
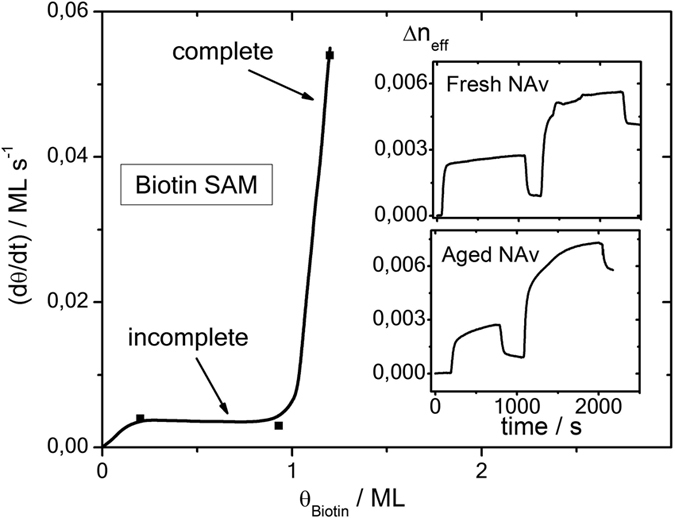
Influence of BIo-SAM coverage on adsorption characteristic of NAv, exploiting the magnitude of the first oscillation. The two insets compare the adsorption feature Δ*n*_eff_(*t*) of aged protein (absence of oscillation) with fresh prepared solution. Oscillations are solely resolved for the non-degraded NAv protein.

The influence of experimental conditions is revealed in Fig. 4. The Fig. 2b,c illustrate the effect of the quality of the attached protein, comparing the degraded NAv solution from Fig. 3b, that was kept as stock solution for 3.7 years around 0 °C, with fresh prepared protein solution from Fig. 3d. With former conditions, oscillatory binding features are completely suppressed, but these are well resolved when high quality protein molecules are used in the experiments. The quality of the pre-deposited BIo-SAM also is crucial for observation of the oscillatory feature. This is illustrated in the Fig. 4, where the magnitude of the first oscillation i.e. the related growth rate *dθ*(*t*)/*dt* is plotted as function of the biotin coverage. At *θ*_Bio_ = 0, oscillations are obsolete, and the final binding state is ascribed to the LA. At *θ*_Bio_ < 1, the growth rate remains at 0.003 ± 0.0005 ML/s and two oscillations are resolved. With *θ*_Bio_ ≥ 1, the oscillation magnitude increases at a factor 20 to 0.06 ML/s and reveals up to 4 oscillations with decreasing magnitudes. Also the binding kinetics of the irreversibly attached NAv layer is about a factor two higher and approaches a final coverage in accord with the RSA mechanism.

**Figure 5:**
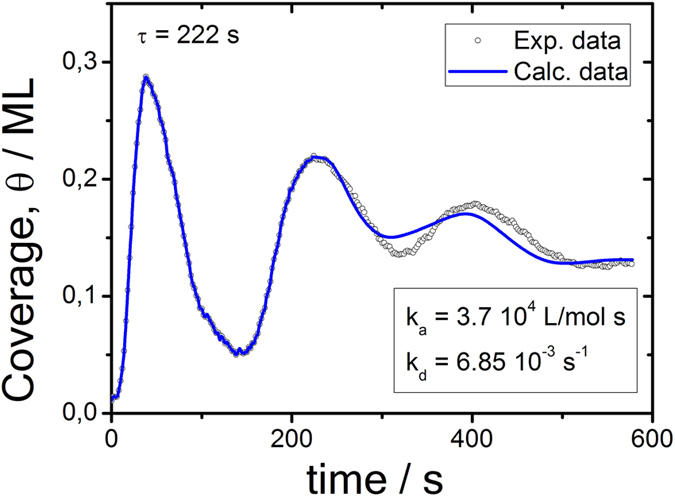
Comparison of experimental data (circles) and numerical plots of theory (blue solid line); applied fit parameters *α, K, R* and delay or residence time *τ*: 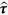  = 222.8337 s, 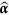 = 0.012263 s^−1^, 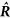 = 0.0016284, 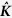 = 1.7896.

Figure 5 illustrates the comparison of the experimentally recorded NAv-coverage *θ*(*t*) with the theoretical model that relies on a numerical integration of equation (7). The steadily increasing background signal, illustrated as the broken line in Fig. 3e comprising a constant growth rate *dθ*(*t*)/*dt*, has been subtracted.

## Estimation Method

To obtain an estimate for the time delay *τ* and the three constants *α*, *K* and *R*, a recursive search procedure is proposed. In this procedure, the values for these parameters are obtained by solving a least-squares problem which is stated in terms of the difference between the experimental data *θ*(*t*) and the predicted data 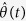. The one step ahead predictor for *θ*(*t*) is obtained by using the forward Euler method to get a discrete-time representation for (7) as given by


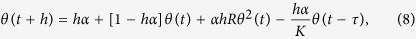


for *t* > *τ*. It is worth to point out that from now on *t* will denote a discrete-time quantity, i.e., *t* = *t*_0_ + (*i* − 1)*h*, *i* = 1, …, *N* in which *h* denotes the sampling interval. Thus, the expression for the 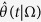 is written as


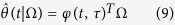


where


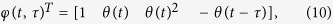



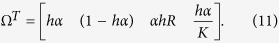


Thus, given *τ*, and for *t* > *τ*, the solution of the parameter estimation problem is stated as


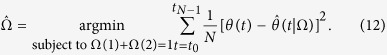


The proposed recursive search procedure consists of the following steps:

Step 0 *k* = 1;

Step 1 Choose the value for *τ*_*k*_, *k* ∈ [1, *M*], *M* ≤ *N*;

Step 2 Estimate the parameters 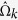 by solving


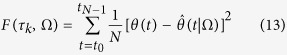



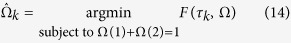


Step 3 Save 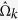, *τ*_*k*_ and 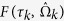 into vector 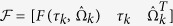 for the *k*^th^ iteration;

Step 5 Set *k* = *k* + 1 and go to Step 1 if *k* ≤ *M*;

Step 6 Sort vector 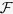 in ascending order of 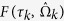 and read the optimal values, i.e., 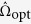 and *τ*_opt_, at the first row;

Step 7 Use the optimal values of 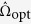 and *τ*_opt_ to compute


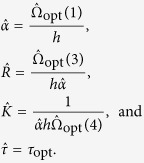


The method allows the determination of the kinetic constants, as well as the delay time 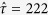 s. This quantity is linked to the surface residence time. Kinetic constants were obtained from best fit parameters as: 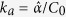 and 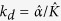 as 3.7 × 10^4^ L/mol/s and 6.85 × 10^−3^ s^−1^ for *h* = 0.4 s, *N* = 1443, and *t*_0_ = 0 s, respectively. The delay time with 222 s is close to, but not exactly the time period between oscillation maxima. Reported oscillation magnitudes significantly exceed those reported in ref. 19.

**Figure 6:**
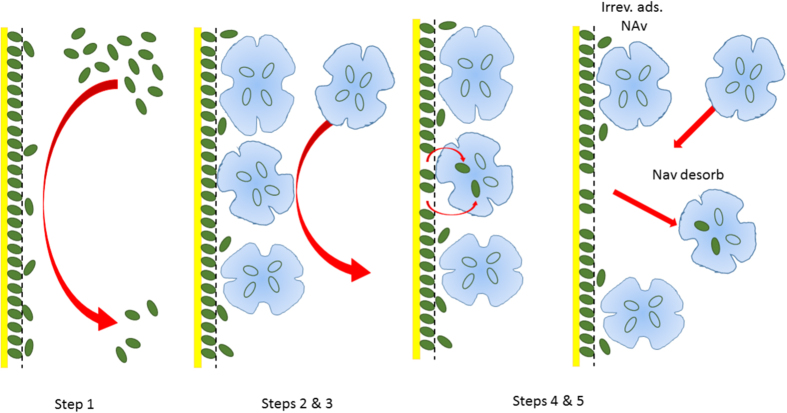
Sketch of the time delayed adsorption process: the quarternary molecular structure of NAv, as taken from Fig. 4 of ref. 21, is applied to the protein symbol. Four voids inside illustrate the four possible biotin positions that define the BIo-NAv complex.

Figure 6 illustrates a sketch of NAv adsorption steps, in presence of time delayed kinetics: 1. Initial biotin adsorption and film formation; 2. Diffusion of NAv molecules to the biotin film; 3. Irreversible NAv attachment onto the surface, possibly with NAv-biotin fraction formation; 4. Simultaneous (irreversible) NAv binding to uncovered fractions on the gold surface and NAv-binding to excess biotin; 5. Delayed desorption of these weakly attached NAv-biotin fractions over time period *τ*, during which the molecules may change their shape (conformational change) or orientation, packing density, undergo chemical surface reactions, change position through surface diffusion including effects of steric hindrance, can be involved. A possible oscillating adsorption process describes as follows: a small amount of attached NAv molecules may re-organize into an “end-on” position, with the open area and adjacent pockets for biotin placed near both ends. This would allow up to two pre-adsorbed biotin molecules to leave the gold surface, since biotin binding to plain gold is relatively weak. They may enter into the empty volume of the NAv molecule, forming a partial BIo-NAv complex, provided that this transition is energetically favorable. Removal of the biotin would suppress the attracting force of the NAv molecule to its previous adsorption site. The particle desorbs from the surface and escapes into the bulk solution. The resulting negative adsorption rate would then cause an oscillation in *θ*(*t*). The strength of this effect varies with, and weakens towards higher coverage, and NAv admission time. It is important to note that this process would be absent in the reverted adsorption system, as mentioned before, and experimentally confirmed (see Fig. 2b,c) Hence, the model suggests that within the framework of the Langmuir approach, an intermediate macro-molecular adsorption state exists between the very short lived reversible and irreversibly states with - theoretically - infinite long residence time.

According to the authors of ref. 18, the adsorption model under consideration aimed at describing the adsorption kinetics of complex molecules at the solid-liquid interface, in presence of conformational structural modifications. It is not fully understood, whether conformational changes apply to the adsorption system under study, since rather expected for irreversibly attached molecules. In an attempt to explore the limitations of the model, the theory has been tentatively applied to small molecule adsorption at the solid-gas interface. As mentioned before, under certain experimental conditions, binding of CO and its oxidation to CO_2_ on Platinum - in presence of oxygen - also leads to periodic kinetic features, which are connected to spatio-temporal self-organization and a catalytic surface reaction. A similar situation - comprising two different reactants - pertains to the surface mediated BIo-NAv complex formation and for both adsorption systems a chemical interaction is involved. The reactive adsorption process at the Pt-metal surface is certainly more complex, since reconstruction and surface transformation processes occur and their dynamic properties are not exactly known. Hence, some arguments to also assume action of a kinetic delay in case of small adsorbing molecules are at hand.

**Figure 7:**
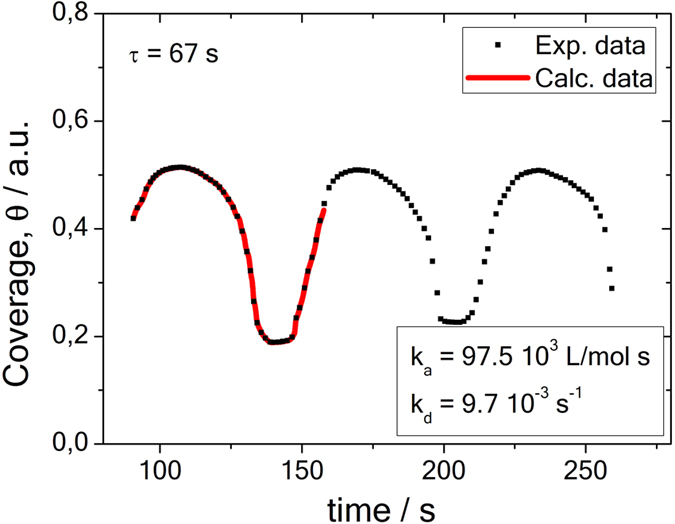
Periodic adsorption pattern for CO-coverage on Pt (110), and comparison with the time delay model (solid red line). Original values of photoemission work function *ϕ* variation from Fig. 3 of ref. 1 associated with and changed to surface coverage values *θ* < 1. Extracted kinetic parameters are: *α* = 1.253 s^−1^, 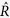 = 0.0040445, 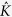 = 247.2515.

Figure 7 exhibits a comparison of the numerically calculated time series data from ref. 1 with the time delay model. For this analysis, the periodic work-function changes from Fig. 3 of ref. 1 have been changed to a surface coverage, assuming that its magnitude is below a single mono-layer. Systematic variations thereof did not result in significant changes of the optimum curve fitting parameters. The calculated delay (or surface residence) time is 67 s and close to the time period of the oscillation. The value is considerably shorter than those obtained for the proteins, whilst kinetic parameters are higher. Most likely, this is due to a stronger, i.e. chemisorptive binding of CO to the oxygen covered Pt substrate beneath.

It is important to mention that the model/concept from refs 18 and 19 are not suited to replace the non-linear dynamics system approach and rate equations of ref. 1, but should be rather considered as a supplement to gain further information on the adsorption system. The time delay and associated surface residence time, respectively, can be understood as the period available to adsorbed molecules to diffuse, react or re-organize, before desorbing or disappearing from the surface. Eventually, this may result in spatial self-organization.

## Conclusions

In conclusion, the present work represents the first report on oscillatory protein adsorption onto a solid substrate. The underlying molecular mechanism supports the adsorption model with delayed kinetics, as published before in refs 18 and 19, which adds the number of established macro-molecular adsorption mechanisms to five. Experimental recordings require well defined initial BIo-SAM formation, as well as high quality protein material and solutions. Apart from kinetic constants and thermodynamic binding properties, the model provides information on the surface residence time, which is otherwise difficult to determine directly. However, it is not associated with or able to provide information on microscopic morphologic features of the binding event. The model has been applied tentatively to reactive small CO-molecule adsorption and well resembles the periodic adsorption characteristics. An extracted residence time can be linked to the dynamics and time scale required for self-organization.

## Additional Information

**How to cite this article**: Neff, H. *et al*. Periodic protein adsorption at the gold/biotin aqueous solution interface: evidence of kinetics with time delay. *Sci. Rep.* **6**, 36232; doi: 10.1038/srep36232 (2016).

**Publisher’s note:** Springer Nature remains neutral with regard to jurisdictional claims in published maps and institutional affiliations.
